# Identification of the Multi-Resistance Gene *cfr* in *Escherichia coli* Isolates of Animal Origin

**DOI:** 10.1371/journal.pone.0102378

**Published:** 2014-07-18

**Authors:** Hui Deng, Jian Sun, Jun Ma, Liang Li, Liang-Xing Fang, Qijing Zhang, Ya-Hong Liu, Xiao-Ping Liao

**Affiliations:** College of Veterinary Medicine, National Reference Laboratory of Veterinary Drug Residues (SCAU), South China Agricultural University, Guangzhou, China; CNR, Italy

## Abstract

Previous study indicated that the multi-resistance gene *cfr* was mainly found in gram-positive bacteria, such as *Staphylococcus* and *Enterococcus*, and was sporadically detected in *Escherichia coli*. Little is known about the prevalence and transmission mechanism of *cfr* in *E. coli*. In this study, the presence of *cfr* in *E. coli* isolates collected during 2010–2012 from food-producing animals in Guangdong Province of China was investigated, and the *cfr*-positive *E. coli* isolates were characterized by PFGE, plasmid profiling, and genetic environment analysis. Of the 839 *E. coli* isolates, 10 isolates from pig were *cfr* positive. All the *cfr*-positive isolates presented a multi-resistance phenotype and were genetically divergent as determined by PFGE. In 8 out of the 10 strains, the *cfr* gene was located on plasmids of ∼30 kb. Restriction digestion of the plasmids with *EcoR*I and sequence hybridization with a *cfr-*specific probe revealed that the *cfr*-harboring fragments ranged from 6 to 23 kb and a ∼18 kb *cfr*-carrying fragment was common for the plasmids that were ∼30 kb. Four different genetic environments of *cfr* were detected, in which *cfr* is flanked by two identical copies of IS*26*, which may loop out the intervening sequence through homologous recombination. Among the 8 plasmids of ∼30 kb, 7 plasmids shared the same genetic environment. These results demonstrate plasmid-carried *cfr* in *E. coli* and suggest that transposition and homologous recombination mediated by IS*26* might have played a rule in the transfer of the *cfr* gene in *E. coli*.

## Introduction

Encoding a methyltransferase that modifies 23S rRNA at A2503, the *cfr* gene confers resistance to five chemically distinct classes of antimicrobials, including Phenicols, Lincosamides, Oxazolidinones, Pleuromutilins and Streptogramin A [1], and also reduces the susceptibility to selected 16-member-ring macrolides such as josamycin and spiramycin [2]. Since its first identification on plasmid pSCFS1 from *Staphylococcus sciuri* [3], the *cfr* gene has been identified on plasmid or chromosome in other staphylococcal species [4], [5], and subsequently in other genera of gram-positive bacteria such as *Bacillus* [6]–[8], *Enterococcus* [9]–[12], *Streptococcus* [13], *Macrococcus* and *Jeotgalicoccus* [14]. In those bacteria, plasmids and various insertion sequences have played an important role in the dissemination of the *cfr* gene between species and genera [15]. In gram-negative bacteria, the *cfr* gene has been sporadically detected in *Escherichia coli* and *Proteus vulgaris* [16]–[18], and little is known about the prevalence and transmission mechanism of the *cfr* gene in these bacteria. Here, we present the first study on the prevalence of the *cfr* gene in *E. coli* isolated from food animals in China. In addition, the main transmission mechanism of the *cfr* gene in *E. coli* was characterized by plasmid and genetic environment analysis.

## Materials and Methods

### Ethics statement

This study protocol was reviewed and approved by the South China Agriculture University Animal ethics committee. The owners of the farm animals from which faecal swabs were taken gave permission for their animals to be used in this study.

### Bacterial strains and Antimicrobial susceptibility testing

A total of 839 *E. coli* isolates were isolated from faecal swabs of diseased food-producing animals submitted to the Veterinary Research Institute, Guangdong Academy of Agricultural Sciences, in Guangdong Province of China during 2010 and 2012 (Table 1). Between three and five herds were sampled from each farm, and all the samples were from 225 farms all over Guangdong province. Bacterial DNA was extracted by a DNA extraction kit (Omega, USA) following the manufacturer's instructions. The presence of the *cfr* gene in *E. coli* was determined by PCR amplification and sequence analysis with the primers described in a previous study [4]. The susceptibilities of *cfr*-positive isolates to ceftiofur, ampicillin, cefotaxime, streptomycin, kanamycin, gentamicin, amikacin, florfenicol, chloramphenicol, tetracycline, ciprofloxacin, enrofloxacin, and trimethoprim-sulfamethoxazole were tested by agar dilution method according to the guidelines of the Clinical and Laboratory Standards Institute (CLSI) [19]. *Escherichia coli* ATCC 25922 was used as the control strain.

**Table 1:** Information on the *E. coli* isolates used in this study.

Year of isolation	Pig			Chicken			Duck		
	No. of faecal swabs	No. of farms	No. of isolates	No. of faecal swabs	No. of farms	No. of isolates	No. of faecal swabs	No. of farms	No. of isolates
2010	264	61	255	105	30	103	96	23	94
2011	89	25	87	57	17	56	42	10	38
2012	124	34	116	41	10	41	51	15	49

### PFGE

Pulsed field gel electrophoresis analysis of *XbaI*-digested genomic DNA of all *cfr*-positive strains was performed using the CHEF-MAPPER System (Bio-Rad Laboratories) as described previously [20]. The PFGE patterns were analyzed with BioNumerics software (Applied Maths, Sint-Martens-Latem, Belgium) using the Dice similarity coefficient with a cut-off at 80% of the similarity values to indicate identical PFGE types.

### Assay of *cfr* transfer

Mating experiments were performed as previously described [21], using azide-resistant *E. coli* J53 or streptomycin-resistant *E. coli* C600 as recipient strain. Transconjugants were selected on tryptic soy agar plates containing florfenicol (10 mg/L) and azide (100 mg/L) or streptomycin (512 mg/L). Plasmid DNA of *cfr*-positive strains was extracted by QIAGEN Plasmids Midi Kit, and was then transformed by electroporation into the recipient strain *E. coli* DH10B. Putative transformants were selected on brain heart infusion agar plates containing florfenicol (10 mg/L).

### Plasmid characterization

The size of *cfr*-carrying plasmid in every parental strain and their transformants was determined using S1-treated genomic DNA followed by PFGE and southern hybridization with probe specific for *cfr* gene. All plasmids from transformants were further analysed by restriction fragment length polymorphism (RFLP) using *EcoR*I (TaKaRa Biotechnology, Dalian, China) restriction enzyme followed by hybridization performed on RFLP gels. Because of the failure in electrotransformation, the *cfr*-carrying plasmid in strain FS13Z3C was analyzed with plasmid extracted from host cell. To investigate the genetic environment of the *cfr* gene, 3 pairs of primers used in previous studies [4], [17], [22] were used for PCR mapping, inverse PCR and sequencing based on the known structure in earlier studies [16], [17].

## Results

### Bacterial strains and Antimicrobial susceptibility testing

Among the 839 *E. coli* isolates, 10 isolates from pig were positive for the *cfr* gene as determined by PCR, which was further confirmed by sequencing the PCR product. Sampling information showed that all the *cfr*-carrying isolates were from different farms except strains 8ZB6D and 8ZG6D which were isolated from different animals of the same farm. Susceptibility testing showed that all the *cfr*-positive *E. coli* strains presented a multiresistance phenotype, including resistance to chloramphenicols, quinolones, ampicillin, kanamycin, gentamicin, tetracycline and trimethoprim-sulfamethoxazole (Table 2).

**Table 2:** Characteristics of the *cfr*-positive *E. coli* strains and the corresponding *cfr*-carrying plasmids.

Strain	Year of isolation	Resistance profile^a^	Phylogroup	*cfr*-carrying plasmid			
				transformation	approximate size (kb)	*EcoR*I fragment(kb)	genetic environment of *cfr* b
FS-P54	2010	CEF,AMP,CTX,STR,KAN,GEN,FFC,CHL,TET,CIP,ENR,SXT	B	+	30	18	A
1ZF13D	2011	AMP,STR,KAN,GEN,FFC,CHL,TET,CIP,ENR,SXT	D	+	30	18	A
2ZX7S	2011	AMP,STR,KAN,GEN,AMK,FFC,CHL,TET,CIP,ENR,SXT	A	+	45	23	B
3ZX12D	2011	AMP,STR,KAN,GEN,FFC,CHL,TET,CIP,ENR,SXT	F	+	30	18	A
8ZG1D	2011	AMP,STR,KAN,GEN,AMK,FFC,CHL,TET,CIP,ENR,SXT	G	+	9,75	9,23	C
8ZB6D	2011	AMP,CTX,STR,KAN,GEN,FFC,CHL,TET,CIP,ENR,SXT	A	+	30	18	A
8ZG6D	2011	AMP,STR,KAN,GEN,FFC,CHL,TET,CIP,ENR,SXT	F	+	30	18	A
8ZG8D	2011	CEF,AMP,CTX,STR,KAN,GEN,FFC,CHL,TET,CIP,ENR,SXT	E	+	30	18	A
8ZG12D	2011	CEF,AMP,CTX,STR,KAN,GEN,FFC,CHL,TET,CIP,ENR,SXT	C	+	30	21	D
FS13Z3C	2012	CEF,AMP,CTX,STR,KAN,GEN,FFC,CHL,TET,CIP,ENR,SXT	H	-	30	18	A

a CEF, ceftiofur; AMP, ampicillin; CTX, cefotaxime; STR, streptomycin; KAN, kanamycin; GEN, gentamicin; AMK, amikacin; FFC, florfenicol; CHL, chloramphenicol; TET, tetracycline; CIP, ciprofloxacin; ENR, enrofloxacin; SXT, Trimethoprim-sulfamethoxazole.

b A, Figure 4a; B, Figure 4b; C, Figure 4c; D, Figure 4d.

### PFGE

**Figure 1:**
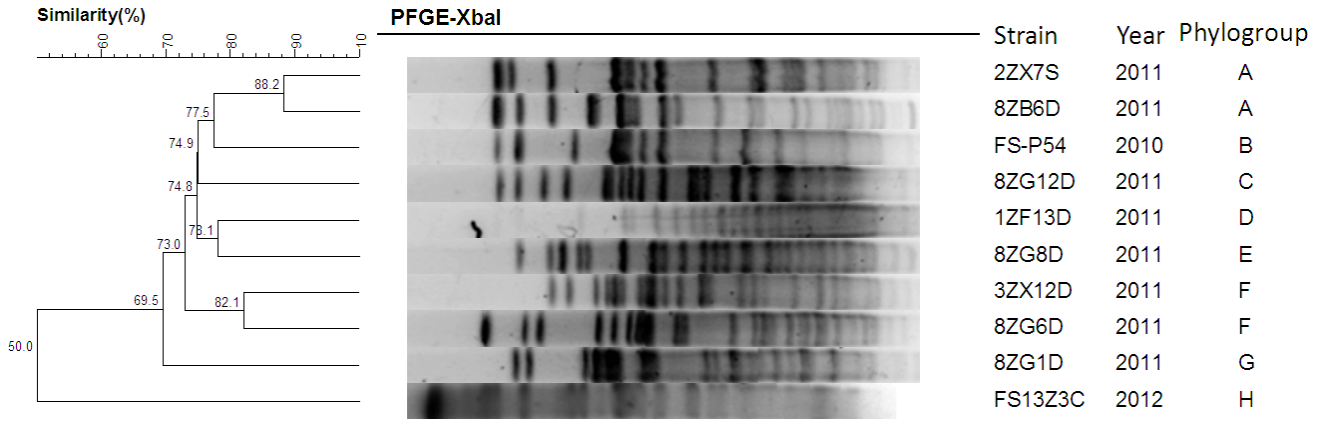
UPGMA dendrogram, PFGE patterns, phylogenetic group and isolation date of *cfr*-positive *E. coli*.

The dendrogram of the *cfr*-positive strains generated from cluster analysis of the PFGE profiles is shown in Figure 1. The 10 strains displayed different PFGE patterns and belonged to 8 phylogenetic groups (designated A to H). Most of the phylogenetic groups comprised a single strain, while Group A and F were represented by 2 strains, respectively.

### Transfer of resistance

The conjugation experiment of *cfr*-positive strains using *E. coli* C600 or *E. coli* J53 as recipient strain failed, but electrotransformation was achieved in all the strains except FS13Z3C. Susceptibility testing of the 9 transformants revealed drastically increased florfenicol MICs (range, 16 to >256 mg/L) compared with DH10B (1 mg/L). Co-transfer of resistance to at least one other antimicrobial was observed in 8 transformants except 8ZG1D-21, which was only resistant to florfenicol. Transformant 8ZG1D-21 was moderately resistant to florfenicol with MIC of 16 mg/L and sensitive to chloramphenicol with MIC of 8 mg/L.

### Plasmid characterization

**Figure 2:**
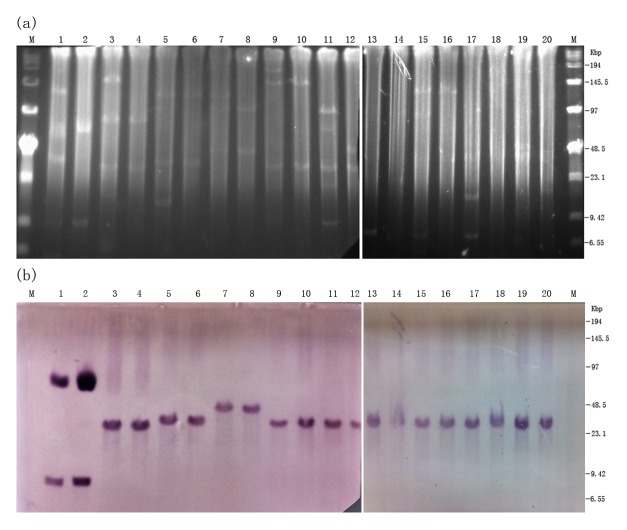
Location of the *cfr* gene in the 10 *E. coli* strains and their corresponding transformants. (a) S1-PFGE of the *cfr*-positive strains and their transformants, (b) subsequent southern hybridization with *cfr*-specific probe. Lanes: M, Low Range PFG Marker; 1, 8ZG1D; 2, 8ZG1D-21; 3, 8ZG12D; 4, 8ZG12D-50; 5, 1ZF13D; 6, 1ZF13D-22; 7, 2ZX7S; 8, 2ZX7S-41; 9, 8ZG6D; 10, 8ZG6D-59; 11, 3ZX12D; 12, 3ZX12D-6; 13, 8ZG8D; 14, 8ZG8D-81; 15 FS-P54; 16,FS-P54-2; 17,8ZB6D; 18, 8ZB6D-30; and 19,FS13Z3C.

**Figure 3:**
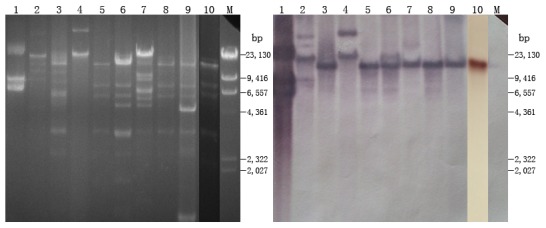
RFLP and hybridization profiles of *cfr*-carrying plasmids in the 9 transformants and strain FS13Z3C. Lanes: M, λ-*Hind*III marker; 1, 8ZG1D-21; 2, 8ZG12D-50; 3 1ZF13D-22; 4, 2ZX7S-41; 5, 8ZG6D-59; 6, 3ZX12D-6; 7, 8ZB6D-30; 8, FS-P54-2; 9, FS13Z3C; and 10 8ZG8D-81.

The result of S1-PFGE revealed that the ten *cfr*-positive strains possessed multiple plasmids of varying sizes, and at least two plasmids were electroporated into the recipient strain. Southern hybridization with *cfr*-specific probe identified the *cfr* gene located on an approximately 30 kb plasmid in all *cfr*-positive strains except 8ZG1D and 2ZX7S (Figure 2 and Table 2). Strain 2ZX7S had a *cfr*-carrying plasmid of ∼45 kb and strain 8ZG1D harbored two *cfr*-positive plasmids that were ∼9 kb and ∼75 kb in size, respectively. The RFLP profiles of *cfr*-carrying plasmids from 9 transformants and the *cfr*-positive strain FS13Z3C were obtained with *EcoRI* digestion and are presented in Figure 3. The plasmids of 5 transformants and the *cfr*-positive strain FS13Z3C showed similar pattern (lanes 3, 5, 6, 8, 9 and 10 in Fig. 3). Although hybridization bands of undigested plasmids due to incomplete enzyme digestion were observed in 3 lanes (Figure 3 lanes 1, 2 and 4), southern hybridization performed on the RFLP gel revealed that the plasmids yielded *cfr*-harboring fragments ranging from 9 to 23 kb, in which a fragment of ∼18 kb was observed in 7 out of the 8 plasmids of ∼30 kb. Additionally, even though the 2 *cfr*-carrying plasmids in 8ZG1D-21 cannot be characterized respectively by RFLP, hybridization showed that the 2 *cfr*-positive plasmids of ∼9 and ∼75 kb yielded *cfr*-carrying fragments of ∼9 and ∼23 kb separately.

### Genetic environment of *cfr*

**Figure 4:**
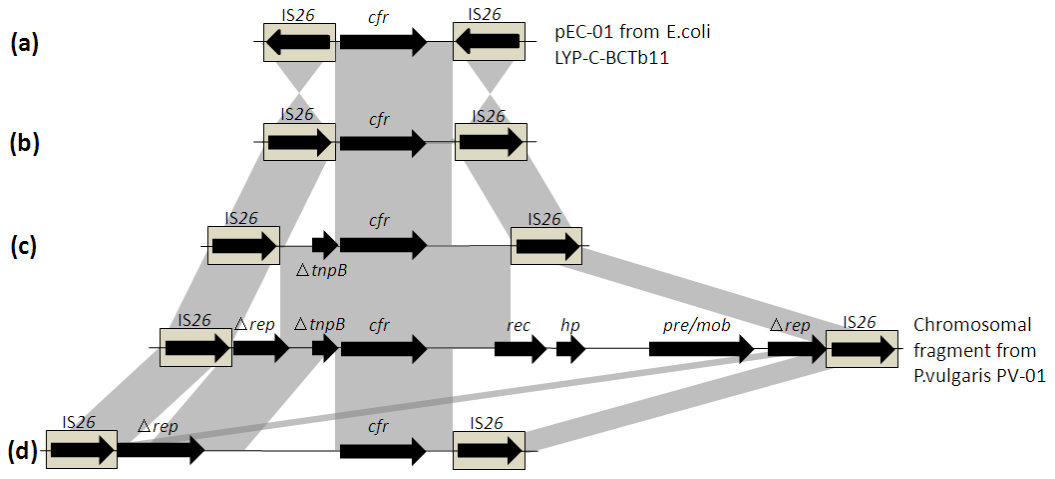
Genetic environment of the *cfr* gene in this study, and structural comparison with plasmids pEC-01 (accession number JN982327) from *E. coli* LYP-C-BCTb11 and Chromosomal fragment from *P. vulgaris* PV-01 (accession number JF969273). The arrows represent the positions and transcriptional direction of the ORFs. The IS*26* elements are shown as light grey boxes. Regions with homology of over 98% are indicated by grey shading. Bacteria corresponding to each genetic environment are as follows: structure a (FS-P54, 1ZF13D, 3ZX12D, 8ZB6D, 8ZG6D, 8ZG8D and FS13Z3C), structure b (2ZX7S), structure c (8ZG1D), structure d (8ZG12D) (see Tables 2).

The result of PCR mapping and sequencing revealed the presence of 4 different genetic environments (Figure 4a-d; GenBank accession number KJ453116, KJ453117, KJ453114 and KJ453115) with the *cfr* gene flanked by two copies of IS*26* located in the same orientation. Among the 8 plasmids of ∼30 kb, 7 plasmids shared the similar genetic environment, in which the *cfr* gene was oriented in the opposite direction of IS*26* (Figure 4a). In contrast, the other 3 strains showed different environments, in which *cfr* was in the same orientation with IS*26* (Figure 4b–d). Structural comparison of the genetic environments showed localized high homology (>98%) with plasmid pEC-01 from *E. coli* LYP-C-BCTb11 and chromosomal fragment from *P. vulgaris* PV-01 [16], [17]. To determine the stability of these structures, inverse PCR was performed, and amplicons of approximately 1570 bp (Figure 4a), 1562 bp (Figure 4b), 2520 bp (Figure 4c) and 4412 bp (Figure 4d) were obtained. Sequence analysis of these amplicons further confirmed the structure of these genetic environments obtained by PCR mapping.

## Discussion

In our study, 10 *cfr*-positive isolates were detected from 839 *E. coli* isolated between 2010 and 2012, and all the positive isolates were from swine farms where florfenicol is extensively used to prevent and cure diseases caused by a variety of bacterial pathogens in China [23]. PFGE analysis of the 10 *cfr*-positive *E. coli* revealed that these strains were genetically divergent. Most of the strains form a distinct phylogenetic group in the dendrogram based on genetic similarity, which suggested that the spread of the *cfr* gene in *E. coli* was not due to clonal dissemination but horizontal transfer.

S1 nuclease PFGE and hybridization showed that the *cfr* gene was located on an approximately 30 kb plasmid in all but two *cfr*-positive strains. Strains 2ZX7S and 8ZG1D harbored *cfr*-carrying plasmids of different sizes. The failure of the conjugation assays using *cfr*-positive strains suggests that the *cfr* gene was carried by nonconjugative plasmids in these strains. Recently *cfr* was identified on a conjugative plasmid in *E. coli* [18], which may further accelerate the dissemination of the *cfr* gene among different Gram-negative bacteria. Although we were not successful in obtaining transformants with a single plasmid after several attempts, RFLP and hybridization profiles of the plasmids showed that 7 *cfr*-carrying plasmids yielded the same-sized *cfr*-harboring fragment of ∼18 kb after digestion with *EcoR*I, and all the 7 plasmids are ∼30 kb in size with the same genetic environments confirmed by PCR mapping and inverse PCR, implying that the 7 plasmids are likely identical and originated from the same source. Interestingly, compared with the 9 strains in which a single *cfr*-carrying plasmid was harbored, strain 8ZG1D and the transformant 8ZG1D-21 have two *cfr*-positive plasmids. Considering the presence of IS*26* flanking the *cfr* gene, IS-mediated recombination may account for the transfer of *cfr* between plasmids in the strain. Interestingly, the result of antimicrobial susceptibility testing showed that 8ZG1D-21 was moderately resistant to florfenicol and sensitive to chloramphenicol, suggesting that the *cfr* gene may confer low-level resistance to chloramphenicols in *E. coli*.

Four different genetic environments were detected surrounding the *cfr* gene, all of which have two copies of IS*26* of the same orientation flanking the *cfr* gene. Previous studies have suggested that insertion element IS*26* can mediate the transfer of *cfr* gene [16], [17]. In our study, structural comparison of the genetic environments showed that part of the segment between IS*26* shares high homology (>98%) with plasmid pEC-01 from *E. coli* LYP-C-BCTb11 and chromosomal fragment from *P. vulgaris* PV-01 [16], [17], further suggesting that IS*26* may have played an important role in the transfer of the *cfr* gene. Furthermore, inverse PCR performed on all of the *cfr*-positive strains can obtain an amplicon, and subsequent sequencing analysis showed that the pair of intact IS*26* flanking the *cfr* gene can loop out the intervening sequence through homologous recombination, which can further accelerate the transfer of *cfr* gene.

To conclude, we present the first study on the prevalence of the *cfr* gene in *E. coli* from food producing animals. The identified *cfr*-positive *E. coli* strains were limited to pigs, coinciding with the extensive use of florfenicol for swine production. PFGE analysis showed that the *cfr*-positive *E. coli* strains were genetically diverse; however, plasmid and genetic environment analysis suggested that most of these strains harbored the same *cfr*-carrying plasmid of ∼30 kb. Additionally, the results suggest that transposition and homologous recombination mediated by IS*26* as well as transformation of *cfr*-carrying plasmids may be the main mechanism for horizontal spread of the *cfr* gene in *E. coli*. Further surveillance and investigation are necessary to facilitate the control of *cfr* spread in gram-negative bacteria.
